# Public’s Mental Health Monitoring via Sentimental Analysis of Financial Text Using Machine Learning Techniques

**DOI:** 10.3390/ijerph19159695

**Published:** 2022-08-06

**Authors:** Saad Awadh Alanazi, Ayesha Khaliq, Fahad Ahmad, Nasser Alshammari, Iftikhar Hussain, Muhammad Azam Zia, Madallah Alruwaili, Alanazi Rayan, Ahmed Alsayat, Salman Afsar

**Affiliations:** 1 Department of Computer Science, College of Computer and Information Sciences, Jouf University, Sakaka 72341, Saudi Arabia; 2 Department of Computer Science, National Textile University, Faisalabad 37300, Pakistan; 3 Department of Computer Science, University of Agriculture Faisalabad, Faisalabad 37300, Pakistan; 4 Department of Basic Sciences, Deanship of Common First Year, Jouf University, Sakaka 72341, Saudi Arabia; 5 Center for Sustainable Road Freight and Business Management, Heriot-Watt University, Edinburgh EH14 4AS, UK; 6 Department of Computer Engineering and Networks, College of Computer and Information Sciences, Jouf University, Sakaka 72341, Saudi Arabia; 7 Department of Computer Science, College of Science and Arts, Jouf University, Qurayyat 77413, Saudi Arabia

**Keywords:** mental health, financial text, machine learning, sentiment analysis, deep learning, the Guardian, support vector machine, AdaBoost, single layer convolutional neural network

## Abstract

Public feelings and reactions associated with finance are gaining significant importance as they help individuals, public health, financial and non-financial institutions, and the government understand mental health, the impact of policies, and counter-response. Every individual sentiment linked with a financial text can be categorized, whether it is a headline or the detailed content published in a newspaper. The Guardian newspaper is considered one of the most famous and the biggest websites for digital media on the internet. Moreover, it can be one of the vital platforms for tracking the public’s mental health and feelings via sentimental analysis of news headlines and detailed content related to finance. One of the key purposes of this study is the public’s mental health tracking via the sentimental analysis of financial text news primarily published on digital media to identify the overall mental health of the public and the impact of national or international financial policies. A dataset was collected using The Guardian application programming interface and processed using the support vector machine, AdaBoost, and single layer convolutional neural network. Among all identified techniques, the single layer convolutional neural network with a classification accuracy of 0.939 is considered the best during the training and testing phases as it produced efficient performance and effective results compared to other techniques, such as support vector machine and AdaBoost with associated classification accuracies 0.677 and 0.761, respectively. The findings of this research would also benefit public health, as well as financial and non-financial institutions.

## 1. Introduction

The definition of health is “a condition of complete physical, mental, and social well-being and not merely the absence of disease or infirmity”. The digital era has brought an unprecedented volume of easily accessible information, including media coverage of current financial events. Daily, the inflection of news articles can elicit emotional responses from readers, and there is evidence of an increase in mental health issues in response to coverage of the recent global financial instability and inflation news [1]. Given the importance and pertinence of this type of information exposure, its daily impact on the general population’s mental health warrants further investigation. Information has economic value because it allows individuals, financial institutions, and government agencies to make decisions that have better-projected payoffs than decisions made without it. Text from digital media (DM), which includes news, events, analyst reports, and public opinions relating to finance, is a substantial source of information that can be used to inform financial policies and decisions. Measuring the targeted information content connected to public attitudes in the text is thus an essential task, not only from the standpoint of the public but also from the perspective of all governmental and non-governmental financial institutions. Due to ambiguities, language variances, syntax, and subjectivity, quantifying the targeted information content of the text can be difficult [2,3].

The proliferation of specialized texts in languages spoken by billions worldwide serves as a standard for information extraction and polarity analysis systems. Using computational linguistics approaches and techniques on documents containing common usage of general language, particularly news stories, public sentiment, whether openly voiced or secretly recorded, is being assessed. Significant changes have occurred in the financial and DM realms due to the rapid expansion of the internet. The rise of DM has posed a challenge to traditional print media, altering people’s jobs and lifestyles. In comparison to traditional media, DM has low costs, great efficiency, broad reach, and high risk [4,5]. Microblogging, e-newspapers, and news channels are examples of new DM that provide financial data. Sentiment analysis and key features of online financial texts assist in determining the public’s sentiment state, offering quick access to public thoughts and attitudes and allowing users to get the information they require quickly. As a result, risk management, public opinion research, and government regulation can all benefit from it [6].

Sentiment analysis or opinion mining is the computational examination of people’s opinions, feelings, assessments, attitudes, moods, and emotions. It is one of the most active research domains in natural language processing (NLP), data mining, information retrieval, and DM mining. Its research and applications have shifted to management and social sciences in recent years, owing to its prominence in general governmental, financial, and social problems. The purpose is to create a structure out of an unstructured natural language text regarding finance and related concepts [7,8,9].

Perceiving emotions is a vital component of human intelligence emulation since it is one of the most fundamental components of personal development and advancement. Not just for the betterment of artificial intelligence (AI), but also the closely related subject of polarity recognition, sentiment analysis is critical [10,11]. The potential to automatically record public attitudes regarding social events, political movements, marketing efforts, and buying patterns has aroused scientific and public interest in the fascinating open challenges in sentimental analysis (SA), categorization, and prediction connected to finance [12]. This has offered to ascend to sentiment analysis, which extracts people’s opinions from ever-increasing volumes of digital data via human–computer interaction (HCI), information retrieval, and multimodal signal processing [13].

Because of the rapid growth of DM platforms, there is now an enormous amount of information on the internet. Users can now share their financial opinions on the internet. User-generated material can be beneficial to businesses at all levels. In this DM era, finding ways to exploit such content becomes critical [14]. Sentiment analysis, often known as opinion mining, is one method of mining user opinion. These two names are sometimes used interchangeably, although they are distinct. Opinion mining is a way of discovering people’s feelings, attitudes, and views regarding a specific topic, whereas sentiment analysis is a way of evaluating people’s opinions, recognizing the sentiment represented in the text, classifying its polarity, and identifying additional sentiment. As a result, sentiment analysis is now regarded as a classification task [15].

Formerly, sentiment analysis was limited to a single domain, but cross-domain sentiment analysis research is currently underway. Previous sentiment analysis research centered on highly subjective texts, e.g., product reviews, movie reviews, and service evaluations, but thanks to the Guardian dataset [16], sentiment analysis has also made its way into newsrooms. The categorization of news is significantly different because the text’s author offers an opinion on product reviews, headlines, and content. In general, news items are objective, and what influences people’s thoughts and sentiments about a specific subject is the text of the reporter or author addressing the issue addressed in the news item, rather than the people’s text [17]. These news items inform various government and financial entities about how the public views and thinks about financial concerns. It can assist them in learning information, such as the quality of their job, the impact of their policies, and their public image. Instead of manually going through all items on DM, the entity in question will benefit from automatically classifying financial material into the appropriate category. The Guardian is regarded as one of the most widely used and well-known DM platforms for up-to-date and accurate financial and government news. As a result, the news in these newspapers occasionally expresses people’s feelings on various themes. Communication and information technologies have significantly impacted the world [18].

Interpersonal relationships, communication patterns, social arguments, political disputes, and DM, for instance, have all altered how people use technology. Political scientists, media & communication experts, sociologists, and experts from the international association have all researched countless stages of social media use [19]. The creative and evolving field of social computing analyses and models social behaviors and events across a variety of platforms. Additionally, this generates innovative and interactive applications that help governmental and financial institutions produce successful outcomes. The reporters or authors of DM can also use the social media content that is available about certain people to express their opinions or feelings on an occasion, problem, or item. It is essential for dissecting this haphazard and inconsistent data to draw conclusions about various topics [20,21]. In addition, the digital platforms that make these data available have a far more formless shape, making mining difficult. The financial text can now be retrieved via a number of DM platforms. One of these is the Guardian application programming interface (API), which gathered text on a certain subject. The following list includes the top four justifications for using The Guardian API [22,23].

- Dissimilar people read The Guardian DM platform for reading news, published daily and presenting the information related to all aspects of life and concerning what people think about the matter under debate, so it is a reliable and reputable source for sentiment or opinion analysis.
- A large number of articles related to financial matters are posted daily in The Guardian newspaper, so it is growing daily.
- The Guardian readers and the consistent users have varied sentiments about diverse topics. Therefore, text posted related to financial matters on DM platform can be collected by using the specific The Guardian API.
- The readers of The Guardian are from all over the world. However, the readers from the United Kingdom prevail over the data that can be collected in English.

Machine learning (ML) methods can be broadly divided into two categories: supervised learning, in which the learning data is presented and provided by the user, and unsupervised learning, in which the learning data is learned as a clustering approach by taking into account the vastness of the dataset [24,25,26,27,28]. Evolutionary algorithms are crucial in this regard; they have been used in a range of optimization tasks, including picture classification, global optimization, text classification, and parallel machine scheduling, to name a few. The arithmetic optimization algorithm is mathematically conceptualized and developed to execute optimization procedures in various search spaces, akin to the reptile search algorithm, a nature-inspired meta-heuristic optimizer influenced by crocodile hunting behavior [29,30,31,32,33].

### 1.1. Motivation

Public feelings associated with finance are gaining significant importance as they help individuals, financial and non-financial institutions, and the government to understand the financial condition, impact of deployed policies, counter-response, and public mental health.

### 1.2. Research Gap

The fundamental problem addressed in this study is to classify public sentiments from the massively available textual dataset based on the financial news primarily published on DM to identify people’s overall views about financial matters that ultimately impact the public mental health.

### 1.3. Objectives

**Figure 1:**
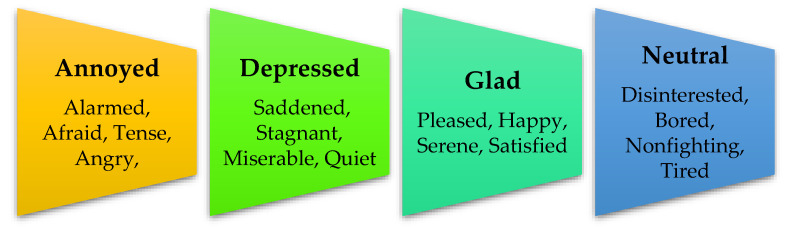
Different Emotional States Adapted from Circumplex Model.

The objectives of the proposed study are as follow:

- Firstly, the dataset collected through The Guardian API is based on public sentiments related to financial news content.
- Secondly, the dataset based on financial news content is preprocessed for appropriate and efficient classification into four primary sentimental attributes: neutral, glad, depressed, and annoyed, selected through the Circumplex model as shown in Figure 1.
- Thirdly, we worked out mainly for the individual sentimental classification with precise accuracy from the mixed state content based on The Guardian dataset (text) using two ML techniques, i.e., support vector machine (SVM) and AdaBoost, and one deep learning (DL) technique, the single layer convolutional neural network (SLCNN).

### 1.4. Contribution of the Study

The study can be helpful for financial, non-financial, and governmental organizations to estimate the impact of their policies through identifying public sentiments that affect public opinion about financial matters and, ultimately, their mental health without direct interaction or survey.

Our goal is to use preprocessed datasets from digital platforms to build an ML-based model for assessing the intended content of financial news. We shall investigate how public perception and mental health via financial news in DM are influenced. Sentiment signals have been employed as a common linguistic characteristic for representing target information content hidden in financial literature. We create a baseline to represent financial news material using frequency-based attributes extracted from emotive words. The ultimate goal of this research would be to create a precise ML-based tool to assist public financial and non-financial institutions.

Numerous investigators employ a variety of ways to categorise various data types as a result of the daily development and expansion of ML methods, including text mining and classification [34,35]. So, for the four groups that do not change frequently in the current study, supervised learning approaches are applied. The preprocessed dataset is required to classify the Guardian-based dataset using ML algorithms and techniques. The Guardian API can access nearly 3085 different contents. Finding the right classification methods is important when the dataset is created in order to classify it. To categorise obtained datasets related to finance, the SVM, AdaBoost, and SLCNN are used because they are capable of handling a high dimensional volumetric dataset [36].

The paper’s organization is as follows. Related research has been explained in Section 2 to highlight the available literature in the related domain, in addition to mathematical models of the deployed ML methods in Section 3. Data collection and feature selection have been explained in Section 4 to describe the whole procedure of preprocessing. Material and methods used have been explained in Section 5 to discuss the procedure of classification and its significance. Section 6 comprises the experimental results and performance comparison of all techniques to highlight their superiority over other existing techniques. Lastly, a discussion and conclusion are presented in Section 7 and Section 8 respectively to conclude the research.

## 2. Related Research

In this section, a literature study is carried out to throw light on attempts of different researchers to enhance understanding of mental health through sentimental analysis and efficient financial text mining and classification. To model and predict people’s sentiments about financial matters can assist public health, financial and governmental organizations in their policy modification and development. Several pieces of research related to the NLP, sentimental analysis, and ML-based algorithms highlight their applications in various fields, proving to be a great source of guidance for the proposed idea.

Using a comprehensive set of NLP techniques and a benchmark of pre-pandemic posts, researchers unearthed patterns of how specific mental health issues emerge in language and recognized at-risk users. Moreover, it evidenced that textual analysis is sensitive to uncovering mental health complaints as they show up in real-time, identifying vulnerable groups and alarming themes during COVID-19, and therefore may be helpful during the continuing pandemic and other world-altering events, such as elections, economic circumstances, and protests [37].

To separate the sentiments from the financial text is done under sentimental analysis. In the sentimental analysis, ML has improved financial text mining, classification, and prediction techniques. The number of publications has grown exponentially to automated sentiment identification through the textual dataset. The text has irregular shapes, and the lexicon makes its sentimental analysis complex and challenging [38,39].

There have been numerous studies on sentiment analysis in the news, with the majority focusing on news stories. Sentiment categorization for online comments on Chinese news was accomplished using supervised ML algorithms. Using Ajax technology, the comments were scraped from SINA [40]. To perform Chinese word segmentation, they employed the ICTCLAS toolset, which includes word segmentation, part of speech tagging, and recognition of unknown terms. Candidate feature determination, feature filtering using information gain, and feature weighing with TF-IDF were all utilized to select features. The SVM and KNN classifiers are utilized. On top stories, SVM outperformed KNN with an accuracy of 60.96%, according to their testing [41].

Another study used common-sense knowledge bases, such as ConceptNet and SenticNet, to develop a sentic computing technique for sentiment analysis of news items from the MPQA corpus. The goal of the study was to use sentiment analysis on individual sentences. A semantic parser, a sentiment analyzer, and a replicated version of the SenticNet database made up the opinion engine. To extract common-sense concepts, each sentence was analyzed semantically. After that, the semantic analyzer linked these concepts to SenticNet sentic vectors. The sentic vector expressed only the feeling described in the statement, not its polarity. Using a polarity measure, the sentic vector was then converted to a polarity score in the range [–1, 1]. The sentic vectors for each thought were derived from the Hourglass of emotions, which classifies sentiments into four categories [42].

The evaluation of Guardian data has been the subject of extensive research in recent years. In their research, the authors suggested a method for dividing student data collected by The Guardian into various groups in order to identify the myriad issues that students face. Therefore, they demonstrated and provided the logical mythos for influencing the emotions expressed on several disparate social media platforms. They have also examined the text and data utilizing advanced annotation, grammar, semantic networks, and vocabulary acquisition. Text classification and basic data gathering methodologies are presented and suggested [43].

The authors have presented research that normalizes irrelevant headlines and content as well as noisy data and categorizes them as positive or negative depending on their polarity. Additionally, they use mixed model approaches to develop phrases that express diverse emotions, and the words they produce are subsequently used as crucial indicators in the classification model. By utilizing various financial message boards, authors have developed a new method for predicting stock market sentiment. They have also automated a projection for the reserve market based on web views [44].

Social computing is a creative and evolving computer model for analyzing and simulating social activities and events across multiple platforms. Using several classifiers, including max entropy and ensembles classifiers, the exactness of the classification technique with a selected characteristic vector is validated for a variety of electrical products. In comparable research, authors have analyzed the classification performance of SMO, RF, NB, and SVM for the Guardian data [45,46].

On social media platforms, satirical news is a common problem that can be false and destructive. The research provided an ensemble technique for identifying satirical news in Turkish headlines. The feature sets were extracted using linguistic and psychological features in the described scheme. Five supervised learning techniques (Naive Bayes algorithm, logistic regression, support vector machines, random forest, and k-nearest neighbor algorithm) were combined with three widely used ensemble methods (AdaBoost, bagging, and random subspace) in the classification phase. Based on the findings, it was determined that the random forest algorithm produced the best results for detecting satire in Turkish. Using the recurrent neural network architecture with an attention mechanism, deep learning (DL) based architectures obtained improved classification accuracy (CA) [47].

Another study used naive Bayes to conduct trials based on various individual attributes obtained from Facebook to predict distinctive personalities. These traits are expressed in the form of English terms that are based on LIWC categories, such as various programs or goals, activity logs, structural networks, and other crucial personal information. Using WEKA, the entire analysis was carried out [48].

Semi-supervised algorithms have also been widely used in the sentiment analysis of news stories. According to the hypothesis, news items can be used to predict real-time changes in stock price direction. Trend segmentation based on linear regression and grouping of intriguing patterns were utilized in their study [49]. They categorized and aligned news articles of importance relative to the trend labels using the semi-supervised Incremental K-Means technique to partition data into two groups. They defined and quantified a cluster discriminating coefficient and a cluster similarity coefficient. To compute a word’s weight in a document, the two metrics were combined with a term frequency score for each word [50]. These properties were employed as input features for ML using a support vector machine on the clustered documents. The positive examples were the clusters of documents that were preserved after preprocessing, while the negative examples were the clusters of documents that were deleted after training two SVM classifiers. The system was tested against a database of around 350,000 financial news stories and the stock prices associated with them, collected from Reuters 3000 Xtra. Their forecasts had a high success rate, indicating they were correct [51].

Due to the vast amount of data accessible, a research group discovered that instance selection and feature selection are critical tasks for attaining scalability in ML-based sentiment categorization. They investigated the effectiveness of fifteen benchmark instance selection procedures in text classification to see how well they could predict the outcome. Decision tree classifiers (C4.5 algorithm) and radial basis function networks regarding classification accuracy and data reduction rates are evaluated, e.g., in terms of selection strategies [52].

According to another study, the vector space model and term weighting approaches are valuable techniques for representing text files. This research thoroughly examined Turkish sentiment analysis, including nine supervised and unsupervised word weighting techniques. The prediction effectiveness of word weighting schemes is investigated using four supervised learning algorithms (Naïve Bayes, support vector machines, k-nearest neighbor algorithm, and logistic regression) and three ensemble learning approaches (AdaBoost, Bagging, and random subspace). The results show that supervised term weighting models beat unsupervised term weighting models in terms of accuracy [53].

According to a group of experts, feature selection is important in constructing strong and effective classification models while lowering training time. They suggested an ensemble feature selection strategy, which combines the distinct feature lists produced by several feature selection techniques to provide a more resilient and effective feature subset. The suggested genetic rank aggregation-based feature selection model is an efficient method that beats individual filter-based feature selection methods on sentiment classification, according to experimental results [36,54].

Researchers demonstrated a DL-based technique for sentiment analysis of Twitter product reviews. The proposed architecture combines CNN-LSTM architecture with TF-IDF weighted Glove word embedding. According to the empirical data, the suggested DL architecture surpasses traditional DL approaches [55].

The review examined the prediction performance of five statistical keyword extraction strategies that used classification algorithms and ensemble methods for scientific text document classification. The paper compares and contrasts five commonly used ensemble approaches with base learning algorithms. The classification schemes are compared in terms of classification accuracy, F-measure, and area under curve values. The empirical analysis is validated using the two-way ANOVA test. Combining a bagging ensemble of random forest with a most-frequent-based keyword extraction technique yields outstanding text classification results, according to the results [56].

According to another article, extracting an efficient feature set to represent text documents is critical in developing a reliable text genre classification scheme with excellent prediction performance. Ensemble learning, which integrates the outputs of individual classifiers to produce a robust classification scheme, is also a prominent research area in ML. An ensemble classification system is proposed based on the empirical study, which merges the random subspace ensemble of random forest with four characteristics (features used in authorship attribution, character n-grams, part of speech n-grams, and the frequency of the most discriminative words). The proposed technique achieved the highest average prediction accuracy for the language function analysis (LFA) database [57].

This research uses supervised hybrid clustering to split data samples of each class into clusters using the cuckoo search algorithm and k-means to give training subsets with greater diversity. The majority voting rule is used to integrate the predictions of individual classifiers after they have been trained on a variety of training subsets. Using eleven text benchmarks, the proposed ensemble classifier’s predicted performance is compared to that of traditional classification algorithms and ensemble learning approaches. The given ensemble classifier outperforms standard classification algorithms and ensemble learning approaches for text categorization, according to the findings of the experiments [58].

The study proposed a sentiment analysis strategy based on ML for students’ evaluations of higher education institutions. They used traditional text representation systems and ML classifiers to assess a dataset of around 700 student reviews written in Turkish. Three traditional text representation systems and three N-gram models were considered in the experimental investigation, along with four classifiers. Four ensemble learners’ prediction performance has also been assessed. The empirical findings show that an ML-based method can improve students’ perceptions of higher education institutions [59].

Another work used traditional supervised learning methods, ensemble learning, and DL principles to develop an effective sentiment categorization scheme with strong prediction performance in Massive open online courses (MOOC) reviews. In the field of sentiment analysis on educational data mining, DL-based architectures outperform ensemble learning methods and supervised learning methods, according to the empirical investigation. In combination with GloVe word embedding scheme-based representation, long short-term memory networks have produced the best prediction performance for all of the compared solutions [60].

The study aimed to develop a methodology for identifying sarcasm in social media data. The authors used the inverse gravity moment-based term weighted word embedding model using trigrams to characterize text documents. By maintaining the word-ordering information, essential words/terms have higher values. A three-layer stacked bidirectional long short-term memory architecture was presented to recognize sarcastic text documents. Three neural language models, two unsupervised term weighting functions, and eight supervised term weighting functions were tested in the empirical study. The provided model performs well in detecting sarcasm [61].

Another study described a DL-based system for detecting sarcasm. In this context, the prediction performance of a topic-enriched word embedding scheme was compared to typical word embedding procedures. In addition to word-embedding based feature sets, traditional lexical, pragmatic, implicit incongruity, and explicit incongruity-based feature sets are considered. The experimental investigation looked at six subsets of Twitter posts, ranging from 5000 to 30,000. The results of the studies suggest that traditional feature sets paired with topic-enriched word embedding approaches can offer promising results for sarcasm detection [62].

For text sentiment categorization, the researchers of this work presented a hybrid ensemble pruning technique based on clustering and randomized search. In addition, a consensus clustering technique is provided to cope with the instability of clustering results. The ensemble’s classifiers are then sorted into groups depending on their prediction abilities. Then, based on their pairwise diversity, two classifiers from each cluster are chosen as candidate classifiers. The search space for candidate classifiers is examined using a multi-objective evolutionary method based on elitist Pareto. The suggested technique is tested on twelve balanced and unbalanced benchmark text categorization difficulties for the evaluation task. In addition, the recommended method is compared against three ensemble approaches (AdaBoost, bagging, and random subspace) and three ensemble pruning algorithms (ensemble selection, bagging ensemble selection, and LibD3C algorithm). The results show that consensus clustering and multi-objective evolutionary algorithms based on Elitist Pareto can be successfully employed in ensemble pruning [63].

A well-known method for understanding and expressing personal opinions, feelings, thoughts, and mental health is text-based sentiment analysis. These people frequently use subjective prose to convey common emotions, moods, feelings, ideas, and reactions. Since most real-world data are amorphous and unstructured, this presents a significant obstacle for emotive analysis. As a result, numerous studies have made impressive attempts to extract meaningful and valuable information from these unstructured and amorphous datasets in recent years. The suggested study is essentially an extension of previous work in the described domain, in which sentimental analysis was performed on a real-time textual dataset pertaining to finance collected via The Guardian API for mental health monitoring. Table 1 presents a summary of the work already done in the identified domain to highlight the area that needs further exploration.

## 3. Mathematical Modeling

Furthermore, we briefly describe the three benchmarked models: SVM, AdaBoost, and SLCNN.

### 3.1. Support Vector Machine

Support vector machine (SVM) is a widely used method that might provide new perspectives on issue solutions [68]. With feature vectors

(1) $$a_{l}\in Y^{m},\;l=1,\;2,\;3,\;\ldots ,\;L\;$$

and binary labels

(2) $$y_{l}\in \;\left\{-1,\;1\right\}$$

Linear classifier defined by

(3) $$v\in Y^{m},\;k\in Y\;:g\left(a\right)=x_{l}^{R}+k\;$$

Perfect separation if

(4) $$y_{l}\geq 1\;\mathrm{for}\;\mathrm{all}\;l\;$$

Otherwise, try to find (v, k) that keeps the classification errors $\delta^{l}$ small.

Usually include in the objective a norm of w or (v, k). The particular choice ${\left||v|\right|}_{2}^{2}$ yields a maximum-margin separating hyperplane.

A famous formulation: SVM (hinge loss)

(5) $$min_{v,k,\delta }=\frac{1}{2}\;{\left||v|\right|}_{2}^{2}+D\sum_{l=1}^{L}max\left(1-y_{l}\left(x_{l}^{R}+k\;\right),0\right)$$

With linear kernel:

(6) $$s\left(t,\bar{t}\right)=t^{T}\;\bar{t}$$

### 3.2. AdaBoost

AdaBoost is an ML approach that stands for “Adaptive Boosting”. The AdaBoost methodology uses a number of classifiers. The sample is split by a previous classifier, which is then used to train a subsequent classifier. This model can increase the adaptability of the AdaBoost approach. The AdaBoost strategy is more resistant to overfitting than other learning techniques in specific classification applications [69]. The classifier used in the AdaBoost method might be inefficient. The voting model states that by determining all of the weak classifiers together, AdaBoost’s classification competence can be improved. With the AdaBoost approach, a weak classifier is improved round by round until it reaches the desired low classification error rate. For each training sample, the weight is given. The weight of this sample and the likelihood that it was chosen by the classifier should both drop if the classifier properly identified this sample. On the other hand, if this classifier mistakenly labels this sample, its weight ought to rise. The AdaBoost technique can therefore focus on the trickier (but insightful) situations.

Let y_j_ be the sample j in the initial sample set S.

D = {y^1^, y^2^, …, y^m^} (7)

$z_{t}^{\left(j\right)}$ means the weight of sample i in k round. The pseudo-code of AdaBoost is as follows:

Initialization:

{

Expert decides maximum round kmax. At first, the weight of each sample is equal.

(8) $$z_{t}^{\left(j\right)}=\frac{1}{m}\;$$

*j* = 1, 2, …, *m*. C_i_ is i^th^ classifier (i = 1, 2, …, n).

}

**Execution**:

For *t* = 1 to n_max_

For i = 1 to n

Set $Err^{t}$ = 0

For *j* = 1 to *m*

{

Use the weak classifier C_i_ to classify sample *j*.

(9) $$Err^{t}=Err^{t}+z_{t}^{\left(j\right)}\;$$

If classifier C_i_ cannot correctly classify sample *j*. Where $Err^{t}$ represents the classification incorrect index of classifier C_i_.

**Calculation**:

{

(10) $$\rho^{t}=\frac{1}{2}\ln\left(\frac{1-Err^{t}}{Err^{t}}\right)$$

$\rho^{t}$ represents the training bias of classifier i. ln() means the natural logarithm function.

{

For *j* = 1 to *m*

}

(11) $$z_{t+1}^{\left(j\right)}=\{\begin{array}{c} z_{t}^{\left(j\right)}\times \exp\left(-\rho^{t}\right),\;if\;classifier\;C_{i}\;cannot\;correctly\;classify\\ z_{t}^{\left(j\right)}\times \exp\left(\rho^{t}\right),\;others \end{array}$$

$\;z_{t+1}^{\left(j\right)}$ means the weight of sample *j* in *t* + 1 rounds. exp() means exponential function.

}

**Output**:

{

(12) $$G\left(Y\right)=argmax_{r}\left(\sum_{i=1}^{n}\rho_{i}^{t_{max}}\times J_{I}\left(s\right)\right)$$

(13) $$J_{I}\left(s\right)=\{\begin{array}{c} 1,\;if\;classifier\;C_{i}\;classify\;i\;as\;s\\ 0,\;others \end{array}\;$$

}

### 3.3. Single Layer Convolutional Neural Network

As illustrated, we use a CNN model with a single convolution layer. Only the vertical spatial relationship is informative since each row of the text processing input matrix represents a dispersed representation of a single word or letter [70]. Rectangular convolution filters with the same width as the input matrix are employed. Only vertical striding is required because the convolution filter and input matrix are the same width. In this structure, the length of an input sentence *n* is a fixed variable and is represented as:

(14) $$t_{1:m}=t_{1}⊕t_{2}⊕t_{3}\ldots \ldots .t_{n}\;$$

where ⊕ is the concatenate operation and $t_{1}$ is the sentence’s lth word. If the sentence is less than the specified length, zero padding is appended to the end of the input matrix. Using successive p words and a filter, the feature *d* is produced using a convolutional process. For instance, the feature *d*_1_ is generated in the following manner:

(15) $$d_{1}=g\left(u\cdot t_{1:m}+c\right)\;$$

where *c* is a bias term, and *u* and *g*(·) denote the convolution filter’s and nonlinear function’s weights, respectively. The feature map is defined by the set dm:

*d* = [*d*_1_, *d*_2_, …, *d*] (16)

Max pooling is applied to *d* for each feature map to obtain the feature map’s maximum value:

(17) $$\hat{d}=max\left\{d\right\}\;$$

**Figure 2:**
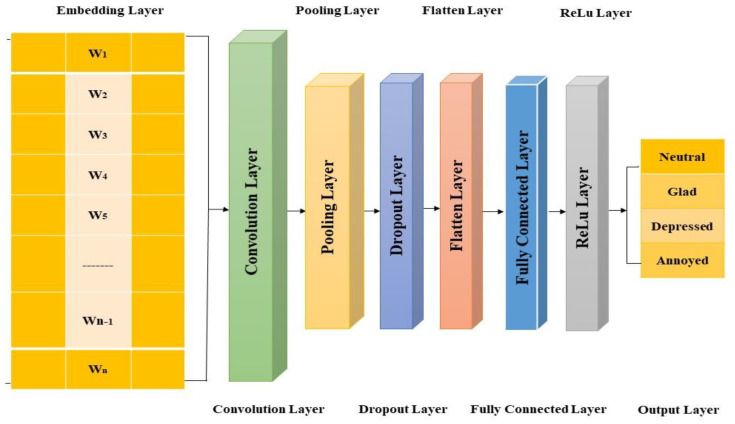
Text-Based Sentimental Classification through Single Layer Convolutional Neural Network.

The textual dataset is represented by a one-dimensional array to the input layer; the kernel has a one-dimensional array format. The outcome of the convolution is called a feature. To find the most important terms related to each convolution filter. A single convolutional layer with a variety of filter sizes was utilized in the experiment. Five filter sizes, 1 × 3, 1 × 7, 1 × 13, 1 × 27, and 1 × 45 are employed, each with 8, 16, 32, 64, and 128 features maps, respectively. After the max-pooling process, a 248-dimensional vector is formed and coupled to the output layer via four nodes: neutral, joyful, depressed, and annoyed. Each feature has an array size of 1 × 1234 due to the usage of zero padding, just like the input text dataset. Each feature has a 1 × 1234 array size and is triggered by the rectified linear unit (ReLU), which is frequently utilized as the activation function for convolutional layers. All activated features (all ReLU results) are concatenated and flattened into a one-dimensional array with a size of 1 × 306,032 in the flatten layer. The flattened data size is then decreased to 1 × 32 sizes by the first fully linked layer, which activates the data using the ReLU. Last but not least, the output layer employs a SoftMax activation function, which is frequently employed in an output layer for trustworthy classification because it offers normalized nonnegative probability values. The output layer has 4 elements (i.e., 1 × 4 array data), which is the same number of sample groups. Figure 2 depicts the structure and process in detail.

Dropout was applied between the final hidden layer and the output layer to regularize the model complexity effectively. Four different word vector techniques are applied while considering the input matrix. The CNN-non-static model uses the word2vec method to fine-tune word vectors after initialization, whereas the CNN-static model does not modify pretrained word vectors. The CNN-rand model was utilized to compare the benchmarked architectures based on an end-to-end learning method. Two sets of word vectors generate a CNN-multichannel model with two channels, and each filter is employed for both channels.

## 4. Dataset Collection and Features Selection

The sentimental analysis and classification based on the financial text published online in The Guardian newspaper are done to identify the sentiments of the people about financial matters. Therefore, it is necessary to collect headlines and detailed news from newspapers through The Guardian API; it fetches Corpus, a collection of documents from The Guardian open platform. The length of the headline and content can vary, but The Guardian delivers a more reliable dataset with complete information and better structure than microblogging platforms. Therefore, the content’s reporter or author must provide the whole message or people’s sentiments by presenting exhaustive information. It is the reason to express the entire scenario related to public sentiments with a comprehensive paragraph because this single paragraph is representative of public opinion or sentiments. The Guardian API offers the capability to retrieve such headlines or paragraphs for a specific topic. We need to insert the API key to proceed with dataset acquisition, then provide the query and set the time frame for retrieving the articles. Further, we must define which features to retrieve from the Guardian platform. Finally, it retrieves the articles. We retrieve 3085 articles mentioning financial text between December 2020 and December 2021. The text will include the article headline and content. In contrast, these article headlines and content are classified into four major sentimental attributes: neutral, glad, depressed, and annoyed.

When the data are collected, the dataset is preprocessed, and unnecessary stuff is removed from these article headlines and content. So, these elements are mandatory for learning the size of that groups. These words are used as elements. The bag of words approach extracts the essential characteristics, and it groups the words from each article headline and content and makes the vector of every article headline and content comprising words. Several researchers have used n-grams in place of these words. Therefore, the grammatical method enhances the position and dimension of the data set. It also uses a unigram, a bigram, and a trigram that compares the pattern. Therefore, these words are selected as features.

In order to generate the dataset, word frequencies were also used. Not all credentials of words are valuable information. Most of the research often avoided prevalent words, did not provide helpful information about the unit and group, and described the utmost general nouns relative to the human language in which most of the text lies. Hence, the general words were isolated from the data set by eliminating high-frequency data. Therefore, the textual dataset’s lowest and maximum cut-off values had to be chosen to find the most refined set of characteristics.

## 5. Materials and Methods

**Figure 3:**
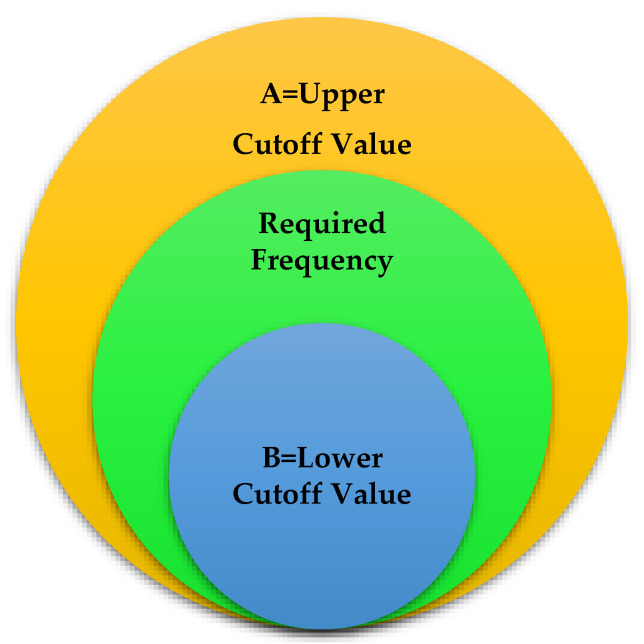
Frequencies Ranges of Chosen Data.

There are two primary learning methods in ML. One is supervised learning and the second is unsupervised learning. The designer provides the system with learning data for training the system in supervised learning, and in unsupervised learning, the system learns the patterns from the data itself. As regards the present situation, the dataset is shapeless and unstructured. The supervised learning mode is much more pertinent. So, the value of A and value of B were selected as frequency-limited, i.e., lower and upper cut-off values that maximize efficiency. Figure 3 depicts the frequency ranges of the selected data.

The proposed work is about text-based sentimental classification using ML techniques: SVM, AdaBoost, and SLCNN. For this purpose, we selected the financial text-based dataset from Guardian containing sentiments. One of the key purposes of this study is to classify public sentiments based on the text primarily used in our daily lives for reporting and writing. Guardian API offers the capability to retrieve relevant headlines and content from articles for a specific query in the specified format. Every query might make more than two thousand text-based results simultaneously. At the same time, these text-based headlines and content are classified into four primary sentimental attributes: neutral, glad, depressed, and annoyed. Proposed attributes and values (text-based) are shown in Table 2.

**Figure 4:**
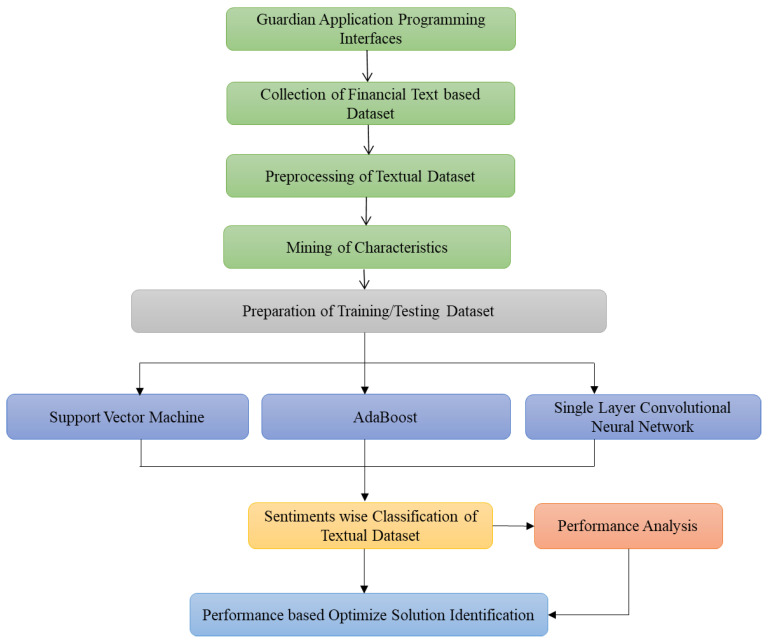
Process for Text-Based Sentimental Classification.

The sentimental classification for mental health monitoring based on the financial text published in the Guardian newspaper online is an emergent area that wants more consideration. Firstly, the Guardian dataset is collected through Guardian API, and then preprocessed. Secondly, the unnecessary stuff is removed from the perfect text, and any feature selection approach is deployed. Thirdly, most data are labeled manually as neutral, glad, depressed, and annoyed headlines and content for preparing the dataset and then its division into two categories training dataset and testing dataset. Finally, the extracted features and their values in the training dataset are used as input to the identified classifiers for modeling and classifying text into the defined four sentiments. Every processing phase is deliberated comprehensively into the below-mentioned subparts. Numerous researchers have also tested various methods for supervised learning, and it has been recognized that the techniques below deliver the most significant and acceptable results compared to other techniques mentioned in the existing literature. Based on their comparative performance for textual data analysis, we have identified three strategies (two ML techniques, SVM and AdaBoost, and one DL technique, SLCNN) in this suggested study. Figure 4 depicts the steps for carrying out the process of financial text-based sentimental classification and mental health monitoring.

The whole research work was carried out in Orange 3.30.2 (University of Ljubljana, Ljubljana, Slovenia). We collected the Guardian dataset from December 2020 to December 2021 using the Guardian API; it contains different types of headlines and content in textual format. In our daily lives, almost everyone reads the newspaper on digital platforms, representing public opinion about domestic or social issues. In this study, we just considered public financial matters gathered through the Guardian API in the form of text. This news consists of neutral, positive, negative, or compound gestures and affects public sentiments. Initially, we preprocessed these headlines and content-based textual datasets and converted them to a refined dataset.

### 5.1. System Specifications

Experiments were conducted on Lenovo Mobile Workstation equipped with Processor: 11th Generation Intel Core i9, Operating System: Windows 11 Pro 64, Memory: 128 GB DDR4, Hard Drive: 2 TB SSD, Graphics: NVIDIA RTX A4000. We used Anaconda Prompt (Jupiter notebook) and Orange-v3.5 tools for the experimentation and results of our proposed scheme, and the language used in it is Python.

### 5.2. Preprocessing

The sentiment dataset was obtained via the Guardian API and contains headlines and content that are neutral, positive, negative, or compound. The anticipated preprocessing scheme divides the text into smaller units (tokens), filters them, performs normalization (stemming, lemmatization), generates n-grams, and labels tokens with part-of-speech labels. The configurations and parameters for preprocessing are listed in Table 3.

### 5.3. Sentiment Analysis

Sentiment analysis forecasts neutral, polar, and compound sentiments for each headline and paragraph in the Guardian newspaper. We used the Vader sentiment modules from Natural Language Toolkit and the Data Science Lab’s multilingual sentiment lexicons. They are all lexicon-based. Vader is only able to communicate in English. Then, using Corpus Viewer, we can view four new features that have been appended to each financial news item via the Vader method: positive score, negative score, neutral score, and compound score. We can see the new features below where the compound was sorted by score. Compound sentiment measures the overall sentiment of financial news, where −1 indicates the most negative sentiment and 1 indicates the most positive sentiment, as shown in Table 4.

**Figure 5:**
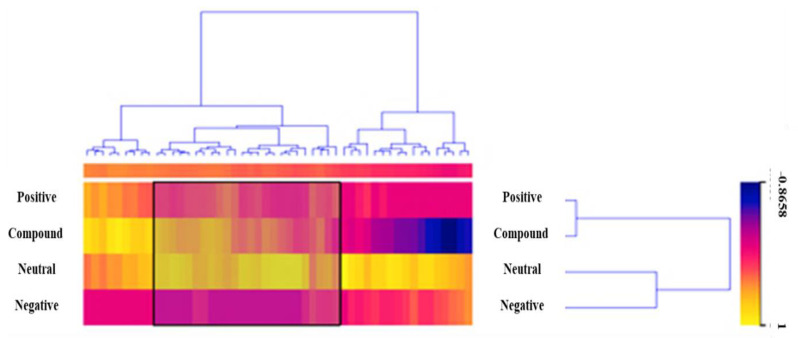
Visualization of Guardian Dataset Through Heat Map.

Now it’s time to visualize the data. We have some features that we are not interested in at the moment, and we will remove them using Select Columns. Due to the fact that we removed News ID via Select Columns. Then we can reduce the size of our dataset to make it easier to visualize. Data Sampler should be used to retain a random 10% of headlines and content. There were 3085 headlines and content related to financial news, but we visualized only 309 using a Heat Map. Now that the dataset has been filtered, it is passed to the Heat Map. Merge by k-means is used to group headlines and content with the same polarity. Rows and columns then cluster the data to create a visualization of similar headlines and content, as illustrated in Figure 5.

### 5.4. Word Cloud

**Figure 6:**
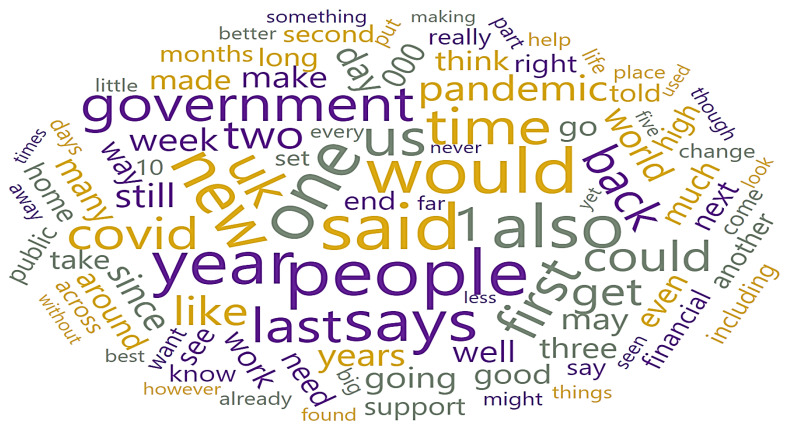
Sentiment Representation through Word Cloud.

As illustrated in Figure 6, a Word Cloud displays tokens from the corpus, with their size denoting the word’s frequency in the corpus or an average bag of words count that summarizes the frequency of use of each word (weight). The outputs contain a subset of the word cloud’s tokens.

### 5.5. Bag of Words

The Bag of Words model generates a corpus of financial news with word counts. The count can be absolute, binary (includes or excludes), or sublinear (logarithm of the term frequency).

It evaluates ML algorithms. Numerous sampling strategies are available, including using distinct test data. It accomplishes two tasks. To begin, it displays a table containing various performance metrics for classifiers, such as classification accuracy and area under the curve. Second, it generates evaluation results those other modules can use to analyze classifier performance, such as ROC analysis or confusion matrix.

### 5.6. Text Mining through Classification Techniques

In this study, SVM, AdaBoost, and SLCNN models were deployed to classify financial news-related sentiments into four categories: neutral, glad, depressed, and annoyed, to monitor mental health. The model was tested for its ability to classify four sentiments. For model evaluation, quantitative measurements, such as area under the curve (AUC), classification accuracy (CA), F1-measure, precision, and recall. The whole process is explained in Algorithm 1:
**Algorithm 1****Input data:**Corpus’s total number of documents within a topic**Transformation of input text**2.Convertion of all text to lowercase3.Removal of accents4.Parsing of HTML tags5.Removal of URLs**Tokenization of text**6.Retention of complete sentences7.Whitespace separation8.Word & Punctuation separation**Normalization through stemming and lemmatization**9.Porter Stemmer application or Snowball Stemmer application10.WordNet Lemmatizer application for a network of cognitive synonyms to tokens11.UDPipe application for normalization of data12.Lemmagen application for normalization of data13.Filteration of words14.Stopwords removal from text15.N-grams range converts tokens to n-grams16.Part-of-Speech tagger performs part-of-speech tagging on tokens17.Averaged Perceptron Tagger uses Matthew Honnibal’s averaged perceptron tagger to perform POS tagging18.Treebank POS Tagger (MaxEnt) performs POS tagging using a Penn Treebank trained model**Term Frequency Identification**19.Term frequency identification to retain most frequent words appears in a document20.Binary checks for the presence or absence of a word in the document21.Sublinear takes the logarithm of the term frequency into account**Classification using SVM, AdaBoost, and SLCNN**22.Sentiment classification

**Figure 7:**
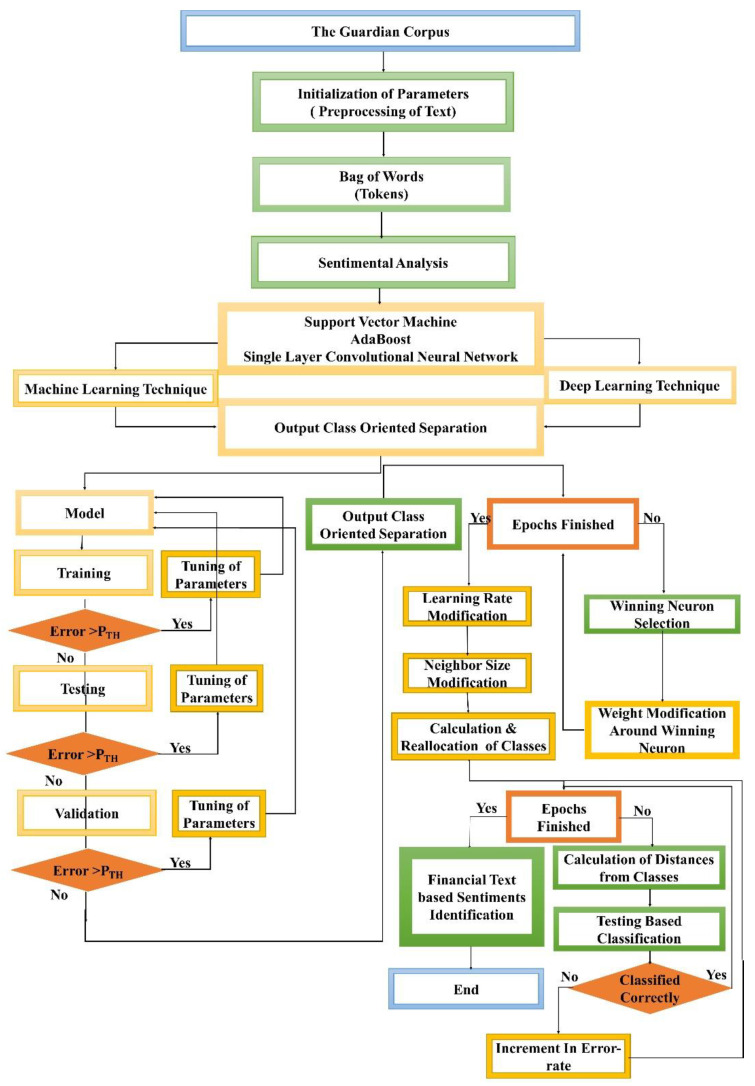
Detail Flowchart of Proposed Work.

Figure 7 summarizes this triad procedure comprehensively, displaying the overall flowchart of the proposed work to illustrate how the system learns through the ML techniques (SVM and AdaBoost) and DL technique (SLCNN) to classify the financial text into the identified sentimental classes. Finally, an optimized mental health identification solution is developed through sentimental analysis of the financial text.

## 6. Experimental Results and Performance Analysis

### 6.1. Financial Text Based Sentiments Classification

SVM, AdaBoost, and SLCNN were deployed to categorize the sentiments using financial text published on DM as we focused on the Guardian newspaper. We processed this dataset through ML techniques, and these data were classified according to four prominent public sentiment attributes: neutral, glad, depressed, and annoyed. The outcome of processed data is shown in Table 5.

**Figure 8:**
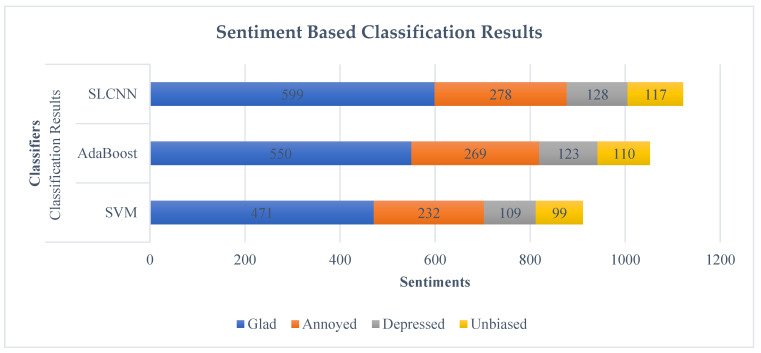
Text-Based Sentimental Classification.

Figure 8 denotes the text-based sentimental classification of Guardian data into four significant sentiment attributes through ML techniques, such as SVM, AdaBoost, and SLCNN. Hence, by analyzing the results, SLCNN provides the best classification result compared to the SVM and AdaBoost.

After the classification of a sentiment-based textual dataset through ML techniques, it is a very significant factor in measuring the accuracy of processed data to check and verify how much given data are classified correctly and how much of the provided data are classified incorrectly. Moreover, accuracy plays a vital role in any process. Table 6 depicted the accuracy measurement among SVM, AdaBoost, and SLCNN of the dataset in the form of how much text data are ideally classified along with the AUC, CA, F1-Measure, Precision, and Recall.

**Figure 9:**
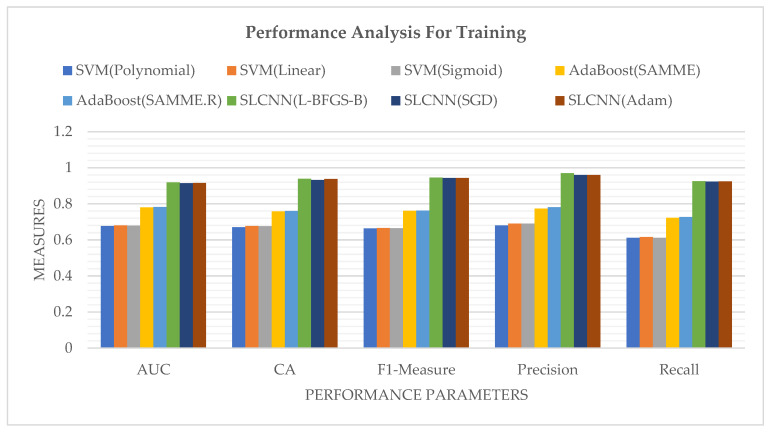
Performance Analysis of SVM, AdaBoost, and SLCNN.

Figure 9 represents the performance measures of SVM, AdaBoost, and SLCNN graphically using the Guardian financial news related dataset into four sentiment classes.

Table 7 indicates the implementation time and the prerequisite of classification techniques for text-based sentimental classification from the Guardian dataset.

**Figure 10:**
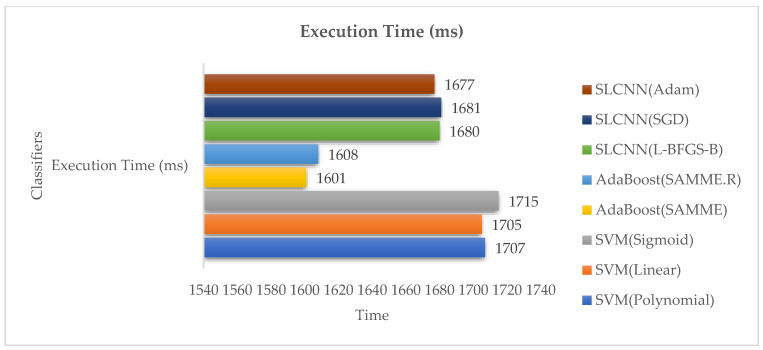
Execution Time Analysis.

Figure 10 indicates the time taken for the implementation procedure. AdaBoost accomplishes the lowest period of execution and implementation time as compared to SVM and SLCNN.

Table 8 indicates the performance measurements of the identified techniques for text-based sentimental classification from the Guardian dataset during testing.

**Figure 11:**
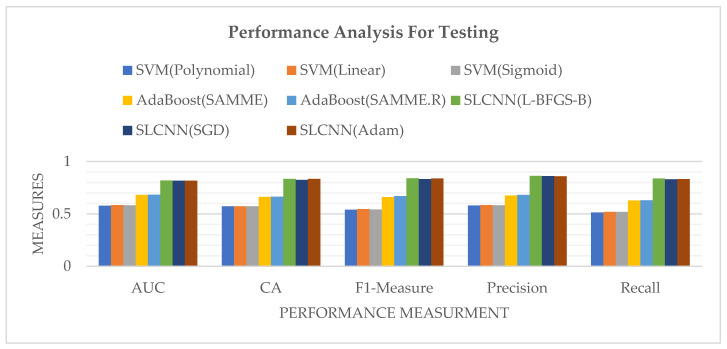
Model Comparison by Performance Measures during Testing.

Figure 11 represents the performance measures of SVM, AdaBoost, and SLCNN graphically using the Guardian financial news related dataset, classified according to four sentiment classes during the testing phase.

### 6.2. Evaluation Metrics Used for SVM, AdaBoost & SLCNN

Various measures are used to determine the execution accuracy of ML methods, and accuracy measures the rate of accurately classified financial text in the respective categories. The classification measures used in our experiments include area under curve (AUC), classification accuracy (CA), F1-Score, precision, and recall. These measures are computed using the following Equations (18)–(22):

AUC = The True Positive Rate (TPR) vs. False Positive Rate (FPR)(18)

TP/TP + FN vs. FP/FP + TN(19)

CA = (TP + TN)/(TP + FN + FP + TN)(20)

F1-Score = (2TP)/(2TP + FN + FP)(21)

Precision = TP/(TP + FP)(22)

Recall = TP/(TP + FN)(23)

**Figure 12:**
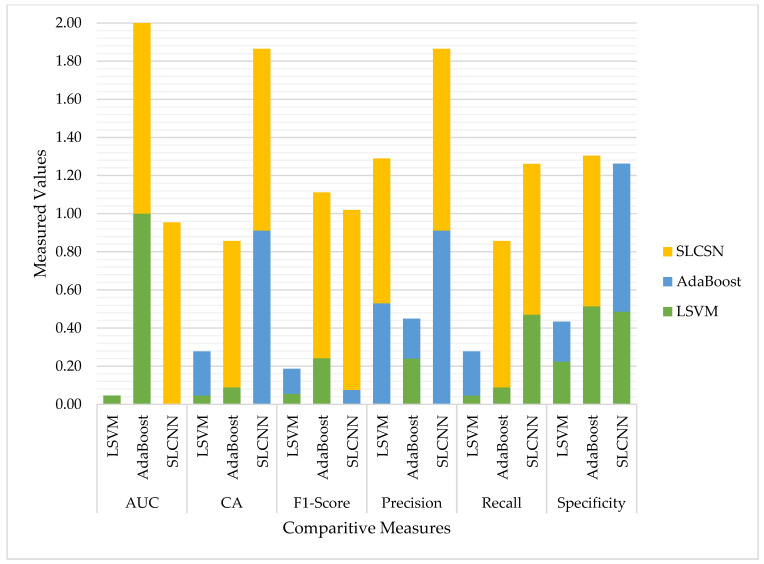
Models’ Comparison for Identified Classifiers.

Finally, Table 9 comprehensively presents the comparative analysis of all three models developed by SVM, AdaBoost, and SLCN. Figure 12 also represents graphically using the Guardian financial news related dataset four sentiment classes classified during the testing phase.

## 7. Discussion

SVM is more compact in memory and computationally efficient due to its single instance training procedure. It can accelerate convergence for larger datasets by utilizing more prevalent parameter tweaks. The steps taken towards the loss function minima exhibit instabilities because of the constant updates, which can cause shifting away from the local minimums of the loss function, and ultimately it may take longer to approximate the loss function minima. Frequent updates use all of the resources at their disposal to process one training sample at a time, thus becoming computationally expensive. This approach does not take advantage of vectorized operations because it only works with one sample at a time. Adaboost is less prone to overfitting than other methods since the input parameters are not simultaneously tuned. Adaboost can be used to increase the accuracy of weak classifiers. Adaboost’s primary disadvantage is that it requires a high-quality dataset. Before implementing an Adaboost algorithm, avoiding noisy data and outliers is necessary. The SLCNN is exceptionally accurate in classifying text. Detects critical traits automatically and without human intervention. Additionally, it is capable of weight sharing and its reliance on initial parameter adjustment (for a good point) enabled SLCNN to avoid local optima. Thus, a shortcoming of SLCNNs is the significant effort needed to initialize them appropriately for the given task. This would necessitate some domain expertise.

In addition, we evaluated the performance of SLCNN in comparison to that of two widely used and well-known ML classifiers. In numerous studies of text classification, DL models have been compared to ML models, but no comparisons have been performed in the literature for financial text classification because DL models have not been employed for sentimental analysis through financial text classification. SLCNN outperforms the other models for two reasons: (a) its multiple filters of varying sizes and structure of hidden layers that captured high-level features from the text; and (b) convolving filters of variable size (window size) can extract variable-length features (n-grams), making it more suitable for financial text classification for mental health monitoring via sentimental analysis. In contrast to ML classifiers, the SLCNN classifier achieves a maximum accuracy of 93.9% on tens of thousands of characteristics from a finance related textual dataset. The primary issue with ML classifiers is that their effectiveness depends on feature selection methods, and comparative studies have shown that no feature selection strategy is effective with all ML classifiers [71].

So, all the above-mentioned pros and cons, along with the experimental results, help us identify an optimized technique for classification using the Guardian-based textual dataset related to financial matters of the public and provide us insights about public mental health. Table 10 compares the performance of the SLCNN and AdaBoost networks to the SVM network for the Guardian dataset containing financial text. In general, increasing the structural complexity of ML models improves sentiment classification performance. To begin, incorporating the SVM and AdaBoost modules enhances classification performance, particularly for financial textual inputs. The AUCs for SVM and AdaBoost were significantly lower than those for SLCNN with textual inputs on the same dataset.

On the other hand, the CA of textual inputs with SLCNN and AdaBoost was generally greater than that of textual inputs with SVM. Additionally, we compared the three models identified using SVM, AdaBoost, and SLCNN using six performance metrics: AUC, CA, F1-Measure, Precision, Recall, and Specificity. The results indicated that SLCNN was more effective than the others in our experiment.

Hence, this is a quantitative analysis of public sentiments obtained through newspaper articles published on DM platforms such as the Guardian in the aftermath of the 2020 COVID-19 outbreak. Using data from the Guardian, we examine how the general public feels about different financial policies and challenges as well as their mental health. We discover that, despite the drawbacks of lockdowns, public opinion is more positive than negative. Although the majority of the headlines and content are considered neutral, the rest is primarily positive. Additionally, the fact that none of the examples have more negative than positive thoughts is comforting. Due to the regulations implemented by financial and political institutions, the results of these assessments can be used to better understand how Guardian users perceive their financial conditions and mental health. The most recent findings offer a starting point for assessing public debate on financial issues as well as recommendations for leading a healthy lifestyle in a period of challenging economic conditions brought on by the pandemic. The information obtained can assist public health officials and policymakers in determining how individuals are coping with financial stress during these extraordinary times and what types of relief should be made available to the public for the betterment of mental health.

## 8. Conclusions, Limitations, and Future Work

SA is a subfield of NLP aiming to classify the sentiment expressed in a free text automatically. It has found practical applications across a wide range of societal contexts, including marketing, economy, public health, and politics. This study aimed to establish the state of the art in SA related to health and well-being by using ML techniques. We aimed to capture the perspective of healthy as well as individuals whose health and well-being are affected, utilizing the available financial dataset of The Guardian newspaper, based on the financial policies of the government and non-government organizations.

The dataset was collected through Guardian API and individual sentiments were classified based on four primary sentiments, i.e., neutral, glad, depressed, and annoyed. We compared three ML based techniques, namely SVM, AdaBoost, and SLCNN, which quickly classified the given text-based dataset into one of four selected individual sentiments. Owing to the daily growth and expansion of ML methods, numerous researchers tend to use these techniques in classifying textual data. SLCNN is considered the best classification method because it has 83.4% accuracy, whereas SVM and AdaBoost have 57.2% and 66.4%, respectively. Hence, it is used when the dependent variable or dependent target is categorical.

The limitations of this investigation include the magnitude of the dataset and the timeframe during which the data were gathered. In order to track how perceptions shift over time, it would be interesting to have data spanning a wider time frame, particularly as the pandemic comes to an end.

Another exciting future path would be to categorize headlines and material according to emotions, such as neutral, glad, depressed, and annoyed, to correctly perceive and reveal the feelings of headlines and content without labeling them.

## Figures and Tables

**Table 1:** Summary of the related work regarding text classification.

References	Techniques	Challenges	Factors Considered	Advantages
[64]	Quad Channel Hybrid Long Short-Term Memory (QC-LSTM) Bidirectional Gated Recurrent Unit (BiGRU)	Huge quantity of detailed information is available in the medical domain, and it is quite a considerable challenge to process it efficiently.	A huge quantity of patient information has been collected in an electronic format.	For medical text classification tasks, machine learning techniques seem quite practical.
[65]	Bidirectional Encoder Representations from Transformers Distil Bidirectional Encoder Representations from Transformers	Training deep language models, however, is time-consuming and computationally intensive.	Financial domain leverage generic benchmark datasets from the literature and two proprietary datasets in the financial technological industry.	Yielded state-of-the-art performance and offload practitioners from the burden of preparing the adequate resources (time, hardware, and data) to train models.
[66]	Support Vector Machines Rocchio classifier	Classification of English text and documents is inefficient.	Size of the feature set.	Better classification of English documents when using more than 4000 features.
[67]	Financial Bidirectional Encoder Representations from Transformers	A limited number of models understand financial jargon.	Financial data for over ten years for 25 different companies.	Can analyze historical data effectively.

**Table 2:** Proposed attributes & possible data types.

Name of Attributes	Possible Data Types
Neutral	Text
Glad	Text
Depressed	Text
Annoyed	Text

**Table 3:** Preprocessing configuration.

**Preprocessor**	**Transformation**	Lowercase
		Remove URLs
	**Tokenization**	Regexp
	**Normalization**	Porter Stemmer
	**Filtering**	Stopwords
		Regexp
	**N-grams Range**	1–2
	**POS Tagger**	Averaged Perceptron Tagger

**Table 4:** Corpus view.

Tweet Content	Positive Score	Negative Score	Neutral Score	Compound Score
On my way home n having.	0.000	0.673	0.337	−0.534
The financial matters and the.	0.235	0.	0.765	0.359
Mmm much better day…	0.848	0.000	0.152	0.445
has work this afternoon…	0.299	0.111	0.590	0.469
--------------------------------				

**Table 5:** Text based sentimental classification.

Sentimental Attributes	Classifiers		
	SVM	AdaBoost	SLCNN
**Neutral**	99	110	117
**Glad**	471	550	599
**Depressed**	109	123	128
**Annoyed**	232	269	278

**Table 6:** Performance measurement during training for text-based sentimental classification (training).

Performance Measures	Classifiers with Optimization Techniques							
	SVM (Polynomial)	SVM (Linear)	SVM (Sigmoid)	AdaBoost (SAMME)	AdaBoost (SAMME.R)	SLCNN (L-BFGS-B)	SLCNN (SGD)	SLCNN (Adam)
**AUC**	0.677	0.680	0.679	0.780	0.783	**0.919**	0.915	0.916
**CA**	0.671	0.677	0.676	0.758	0.761	**0.939**	0.932	0.938
**F1-Measure**	0.664	0.666	0.665	0.762	0.763	**0.946**	0.944	0.943
**Precision**	0.681	0.691	0.690	0.774	0.782	**0.970**	0.960	0.960
**Recall**	0.612	0.616	0.612	0.723	0.727	**0.926**	0.924	0.925

**Table 7:** Execution time of the Guardian dataset analysis for text-based sentimental classification.

Machine Learning Techniques	Execution Time (ms)
**SVM (Polynomial)**	1707
**SVM (Linear)**	1705
**SVM (Sigmoid)**	1715
**AdaBoost (SAMME)**	1601
**AdaBoost (SAMME.R)**	1608
**SLCNN (L-BFGS-B)**	1680
**SLCNN (SVM)**	1681
**SLCNN (Adam)**	1677

**Table 8:** Performance measurement during testing for text-based sentimental classification (testing).

Performance Measures	Classifiers with Optimization Techniques							
	SVM (Polynomial)	SVM (Linear)	SVM (Sigmoid)	AdaBoost (SAMME)	AdaBoost (SAMME.R)	SLCNN (L-BFGS-B)	SLCNN (SVM)	SLCNN (Adam)
**AUC**	0.578	0.583	0.581	0.680	0.682	**0.819**	0.817	0.817
**CA**	0.571	0.572	0.572	0.661	0.664	**0.834**	0.824	0.833
**F1-Measure**	0.540	0.546	0.541	0.659	0.669	**0.840**	0.831	0.837
**Precision**	0.579	0.583	0.582	0.675	0.680	**0.863**	0.861	0.858
**Recall**	0.512	0.519	0.518	0.627	0.629	**0.837**	0.829	0.831

**Table 9:** Models’ comparison by performance measurements.

Model Comparison.	Machine Learning Techniques	SVM	AdaBoost	SLCNN
**Area Under Curve**	**SVM**	0.046	0.000	
	**AdaBoost**	1.000		1.000
	**SLCNN**		0.000	0.954
**Classification Accuracy**	**SVM**	0.046	0.232	
	**AdaBoost**	0.089		0.768
	**SLCNN**		0.911	0.954
**F1-Score**	**SVM**	0.056	0.131	
	**AdaBoost**	0.242		0.869
	**SLCNN**		0.0758	0.944
**Precision**	**SVM**		0.530	0.760
	**AdaBoost**	0.240	0.209	
	**SLCNN**		0.911	0.954
**Recall**	**SVM**	0.046	0.232	
	**AdaBoost**	0.089		0.768
	**SLCNN**	0.470		0.791
**Specificity**	**SVM**	0.224	0.210	
	**AdaBoost**	0.514		0.790
	**SLCNN**	0.486	0.776	

**Table 10:** Performance comparison of the anticipated text classification models.

	Classifier	AUC	CA	F1-Score	Precision	Recall
**Aloqaily et al. [72]**	Logistic Model Trees	-	0.8550	-	-	-
**Prasetijo et al. [73]**	SGD Modified Hurbe	-	0.8200	0.6435	-	1.00
**Prasetijo et al. [73]**	SVM	-	0.784	-	0.548	1.00
**Asghar et al. [74]**	BiLSTM	-	0.8766	0.8766	0.8766	0.8766
**Alanazi et al. [75]**	ODCNN	0.990	0.998	-	-	-
**Asghar et al. [76]**	BiLSTMATTN	-	0.9286	0.93	0.93	0.93
**Anticipated (Financial Text based Sentiments’ Classification)**	SVM	0.583	0.572	0.546	0.583	0.519
	AdaBoost	0.682	0.664	0.669	0.680	0.629
	SLCNN	0.819	0.834	0.840	0.863	0.837

## Data Availability

The dataset used in this study is publicly available.
